# Automatic selection of representative proteins for bacterial phylogeny

**DOI:** 10.1186/1471-2148-5-34

**Published:** 2005-05-31

**Authors:** Marshall Bern, David Goldberg

**Affiliations:** 1 Palo Alto Research Center, 3333 Coyote Hill Road, Palo Alto, CA 94304, USA

## Abstract

**Background:**

Although there are now about 200 complete bacterial genomes in GenBank, deep bacterial phylogeny remains a difficult problem, due to confounding horizontal gene transfers and other phylogenetic "noise". Previous methods have relied primarily upon biological intuition or manual curation for choosing genomic sequences unlikely to be horizontally transferred, and have given inconsistent phylogenies with poor bootstrap confidence.

**Results:**

We describe an algorithm that automatically picks "representative" protein families from entire genomes for use as phylogenetic characters. A representative protein family is one that, taken alone, gives an organismal distance matrix in good agreement with a distance matrix computed from all sufficiently conserved proteins. We then use maximum-likelihood methods to compute phylogenetic trees from a concatenation of representative sequences. We validate the use of representative proteins on a number of small phylogenetic questions with accepted answers. We then use our methodology to compute a robust and well-resolved phylogenetic tree for a diverse set of sequenced bacteria. The tree agrees closely with a recently published tree computed using manually curated proteins, and supports two proposed high-level clades: one containing Actinobacteria, Deinococcus, and Cyanobacteria ("Terrabacteria"), and another containing Planctomycetes and Chlamydiales.

**Conclusion:**

Representative proteins provide an effective solution to the problem of selecting phylogenetic characters.

## Background

In molecular phylogeny, a great deal of attention has gone to computational methods for building phylogenetic trees [1], but much less to methods for selecting phylogenetic characters. Most sequence-based studies of prokaryotic and universal phylogeny have used either small-subunit rRNA genes [2-4] or highly conserved proteins such as ribosomal proteins, elongation factors, chaperones, and tRNA synthetases [4-6], arguing that these core sequences are unlikely to be horizontally transferred and hence should reflect vertical descent. Another sequence-based method relies upon the presence/absence of hand-picked "signature sequences" (conserved insertions/deletions) [7,8] to infer descent. This method does not specifically handle horizontal transfers, but can sometimes resolve a short internal branch that cannot be unambiguously resolved by a continuous evolutionary model. Early phylogenetic studies [2,3,8], limited by availability, were forced to use manually selected characters, but with the recent proliferation of full bacterial genomes, this restriction no longer applies, as all genes and genome data have now become potential characters.

Whole-genome phylogeny has the potential to discern vertical descent even in the case of widespread horizontal transfer. Many recent attempts at whole-genome phylogeny have used automatically computed characters other than sequence, such as gene order [6], dinucleotide frequencies [9], presence/absence of orthologous pairs [10], and presence/absence of gene families [11,12]. These non-sequence studies have given bacterial phylogenies with substantial areas of disagreement, and indeed it has been found that for deep prokaryotic phylogeny, sequence generally carries a stronger signal than dinucleotide frequencies [9], gene order [13], or number of common orthologs [6]. Hence there arises a need for a phylogenetic methodology that combines the power of sequence-based approaches with the objectivity and completeness of whole-genome approaches.

Researchers have responded to this need with a number of approaches that seek a predominant set of "concordant" [14] genes compatible with the same phylogeny. The approaches vary in whether they use gene trees or distance matrices to evaluate the genes, and also in their levels of automation and completeness. The use of gene trees is more common than the use of distance matrices. Brochier et al. [4] start with 57 translational apparatus proteins, ubiquitous over the set of organisms under study. They automatically screen these genes to obtain 44 concordant genes by principal component analysis of vectors of likelihoods for 375 test tree topologies; they associate the first principal component with gene length and the second with "incongruence". Along similar lines, Zhaxybayeva and Gogarten [15,16] evaluate genes by probability mapping of small trees such as four-taxon "quartets". Battistuzzi et al. [17] manually curated 60 ubiquitous COGs [18] down to 32 by rejecting all those that gave unstable gene trees, gene trees with either archaebacteria or eubacteria non-monophyletic, or "deep nesting" of a species from one phylum within another phylum. Daubin et al. [19] cluster the topologies of 310 computed gene trees in order to find a concordant set of 121, and then combine the concordant trees using the "supertree" approach [20-22], which can accommodate the missing data resulting from non-ubiquitous genes.

The use of organismal distance matrices to evaluate genes is less common, but as we argue below, it has a number of advantages over the use of gene trees. Clarke et al. [14] and Gophna et al. [23] compute distance matrices from reciprocal-best BLAST scores on a large number of genes. They measure the concordance of genes by correlation with the median distance matrix. They then use the consensus distance matrix of the concordant genes [14] or a weighted combination of the gene distance matrices [23] to compute the tree directly, rather than employing a more principled, model-based method such as maximum likelihood [1,24,25]. Novichkov et al. [26] improve upon the distance matrices of Clarke et al. by using a linear measure of evolutionary distance rather than BLAST scores and by assigning rate parameters to correct for the differing mutability of genes. They do not, however, go on to compute phylogenies based on concordant genes.

In this paper, we introduce and validate a fully automatic, whole-genome methodology based on "representative sequences". We start by assembling all of the highly conserved proteins (families of orthologs) within the set of genomes. We then use these genes to compute a consensus distance matrix by an algorithm similar to that of Novichkov et al. [26]. We use the consensus distance matrix to select representative sequences, but not to build the phylogenetic tree directly. Representative sequences are contiguous subsequences – typically 300 residues – from ubiquitous, conserved proteins, such that each orthologous family of representative sequences taken alone gives a distance matrix in close agreement with the consensus matrix. The phylogenetic tree is then computed using maximum-likelihood methods on a multiple alignment of a concatenation of representative sequences. We validate the methodology on a set of small phylogenetic problems with accepted answers, before going on to compute automatically a phylogeny for all of Bacteria. Our overall bacterial phylogeny shows striking agreement with the tree produced by Battistuzzi et al. [17] using genes that were manually curated for agreement with accepted clades.

We chose to use distance matrices rather than gene trees for the evaluation of gene concordance, because trees are brittle: a small change in sequence can dramatically change tree probabilities or the topology of the likeliest tree. (Even in continuous tree-space [27], small changes in input can force large changes in trees if the sequence data is not consistent with any one tree.) Moreover, distance matrices can more easily incorporate missing data: each sufficiently conserved gene, ubiquitous or not, can contribute to the pairwise distances between the organisms containing that gene. Combining trees on different sets of organisms is not as straightforward; indeed supertree methods typically combine matrix encodings [20-22]. Our method is conservative in its use of non-ubiquitous genes, including only those well-conserved proteins appearing in at least three-fourths of the taxa under study, but even at this level it can typically use more than three times as many genes as are completely ubiquitous over the taxa. Finally, anomalous pairwise distances directly locate likely horizontal gene transfers and other discontinuous evolutionary events (such as large insertions or deletions, loss of a domain, hidden paralogy, or rapid evolution due to change of function), and indeed our method rediscovers a number of previously proposed horizontal transfers.

## Results

Our computational experiments produced: (1) an evaluation of the methodology of representative proteins, (2) findings concerning which proteins make the best phylogenetic characters, and (3) a fairly complete and well-resolved phylogeny for sequenced Bacteria.

### Methodology

We evaluated our methodology on a test set of 10 deep phylogeny problems with known answers, comparing a maximum-likelihood tree-building method using representative sequences with the same method using an identical amount of randomly chosen, highly conserved, ubiquitous protein sequence. The same data-mining program compiled the families of orthologs in each case, but the randomly chosen proteins were picked without regard to their "representativeness", and hence are equivalent to small subsamples from all highly conserved ortholog families. We used small subsamples, because concatenations of 20 or more highly conserved genes almost invariably give correct trees for our relatively easy test set problems. As seen in Table 1 (also see Table 3), representative proteins generally outscore randomly chosen proteins in number of correct single-gene trees, number of accepted clades found over all gene trees, and number of correct trees on concatenations of genes. The consensus gene tree (that is, consensus over all single-gene trees) made with representative proteins succeeded (included all accepted clades) on all problems except 4 and 9, and a consensus (over bootstraps) concatenated tree succeeded on all problems except 4. Randomly chosen proteins succeeded less often. The consensus gene tree made with randomly chosen proteins failed on problems 1, 2, 7, and 9, and the consensus concatenated tree failed on problems 1, 2, 7, 9, and 10. The one problem on which randomly chosen ubiquitous proteins outscored representative proteins was problem 4, for which representative proteins often divided the organisms as ((Buchnera, Rickettsia), Mycoplasma) (Staphylococcus, (Mycobacterium, Bifidobacterium)), not finding the relatedness of Mycoplasma and Staphylococcus. As intended, representative proteins accurately represent the consensus distance matrix, which includes the contributions of many non-ubiquitous genes and has large distances to the endocellular organisms Buchnera and Mycoplasma, whereas the randomly chosen ubiquitous genes underrepresent the genomic distances to these two organisms and thus do not imply a tree with such long branches. Altogether the results on the 10 test problems suggest that representative proteins reconstruct short internal branches more efficiently than do randomly chosen ubiquitous proteins, but at the cost of greater susceptibility to long-branch attraction.

**Table 1:** Validation of our methodology on 10 deep phytogeny problems. Organism abbreviations are shown in Table 3, and the accepted clades are shown with parentheses. The column labeled "# Clades" gives the number of accepted clades to be found. The column labeled "# Genes" gives the number of genes used. The Trees column gives the number of gene trees that find all the accepted clades; results for representative proteins are on the left, and results for randomly picked ubiquitous proteins are on the right. For each gene, the most conserved 300-residue sequence was used, and randomly picked proteins were matched to the representative proteins in overall conservation level. Consensus gives the number of accepted clades found over all gene trees; an asterisk indicates that the consensus tree (computed using CONSENSE from the PHYLIP package [52]) finds all the accepted clades. Concatenation gives the number of clades found in 100 bootstraps from a concatenated alignment of all genes; an asterisk here indicates the success of the consensus over bootstrap trees. In problem 6 for example, there are 5 accepted clades, 8 single-gene trees, and 100 bootstrap trees, so a perfect "Consensus" score would be 40, and a perfect "Concatenation" score would be 500.

Organisms	# Clades	# Genes	Trees		Consensus		Concatenation	
1. (Borr, Trep) (Chlor, Bac) (Campy, Bruc)	3	8	8*	2	24*	12	299	112
2. (Neiss, Rals) (Xyl, Haem) (Rick, Meso)	3	8	5*	3	21*	19	247	207
3. (Clost, Lacto) (Mycob, Bifid) (Campy, Rick)	3	8	6*	4*	18*	18*	294	283
4. (Buch, Rick) (Mycob, Bifid) (Staph, Mycop)	3	8	2	1*	13	15*	235	297
5. (Urea, Mycop) (Strep, Lacto) (Staph, List)	3	8	8*	5*	24*	21*	300	300
6. (Syn, Pro) (Rick, Buch) (Chlor, Bac) (Staph, Strep) (Borr, Trep)	5	8	7*	2*	37*	26*	481	472
7. ((Rick, Bruc) ((Vib, Esch, Haem), Neiss) (Heli, Campy)) (Syn, Pro) (Clost, Staph) (Borr, Trep)	8	17	3*	3	129*	108	762	741
8. ((Caul, Meso), Esch) (Chlor, Bac) (Pro, Nos)	4	8	7*	3*	30*	27*	400	398
9. ((Geo, Desulf), (Wol, Campy), (Caul, Rick)) (Borr, Lep) (Chlor, Bac)	6	8	1	2	31*	32	554	512
10. (Chlor, Bac) (Mycop, Strep, Clost) (Mycob, Bifid)	3	8	1*	2*	15*	13	255	245

### Proteins as phylogenetic characters

**Figure 1:**
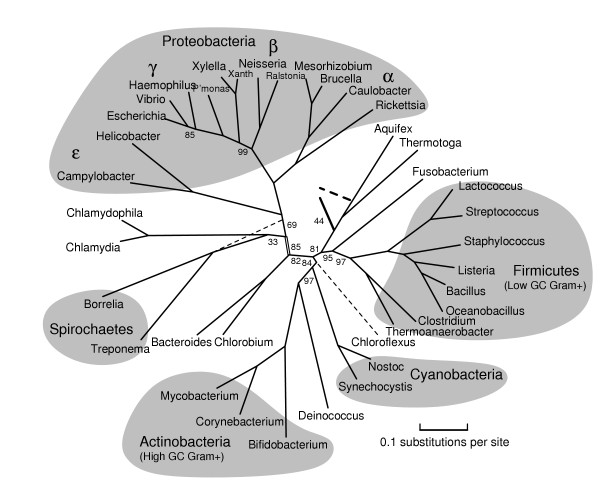
**A rooted phylogenetic tree of Bacteria computed from representative proteins**. As explained in the text, this tree was computed first altogether, then with an outgroup of Aeropyrum and Methanopyrus to place the root, and then again in overlapping halves using different proteins, with the split at the doubled edge near Chlorobium. The numbers indicate bootstrap support for clades out of 100 trials; omitted numbers are all 100. The bootstrap support for the root is 44; the second choice is shown dashed. The weakest bootstrap support is for the Spirochaetes and Chlamydiales clade; again the second choice is shown dashed. The Chloroflexus genome is available only in a draft; we give it a tentative placement, without bootstrap support or edge lengths, based on about 1200 columns.

We find that protein representativeness cannot be determined a priori, as many favored proteins such as elongation factors and ribosomal subunits turn out to be poor representatives for certain sets of genomes. For example, EF-G is a relatively weak representative for Proteobacteria; the pairwise distance matrix computed from EF-G alone has a correlation coefficient of .64 with the 200-protein consensus distance matrix, whereas the best proteins such as GroEL have correlation coefficients greater than .90 as shown in Table 2. Yet EF-G is an acceptable protein for rooting the bacterial tree or for computing the initial, overall tree for Figure 1 using a diverse set of 30 bacteria; for these tasks its correlation coefficients of .77 and .70 are not much lower than those of the best proteins. Brochier et al. [4] also found EF-G suspect; it fell outside of their main cluster, and with further analysis they found probable horizontal gene transfer (HGT) between *β*- and *γ*-Proteobacteria. Ribosomal proteins S1, S14, and L4 are poor representatives for the diverse set of 30 bacteria, with anomalously small distances between Deinococcus and *α*-Proteobacteria, Actinobacteria and Proteobacteria (also noted by [28]), and Actinobacteria and Firmicutes, respectively. Yet S1 turns out to be a good representative for Proteobacteria alone; in this case S1 has a correlation coefficient of .88, whereas for the diverse set the correlation coefficient is only .29. Generally tRNA synthetases are less representative than elongation factors and ribosomal subunits.

**Table 2:** Representative proteins used to compute Figure 2. Class is the COG functional code [18]. Rank is rank in a list of most conserved proteins (families of orthologs), from 0 to 199, for the set of genomes under study; thus FtsH (rank 2) is more conserved than DNA polymerase I (rank 59). Coeff, S. Dev, and Max are respectively the correlation coefficient, the standard deviation, and the maximum elementwise difference between the scaled distance matrix given by this protein and the consensus distance matrix. Distances for this set of organisms were approximately 0–150. Each sequence was limited to the most conserved 300-long amino acid sequence for the protein.

Gene	Class		Name	COG	Rank	Coeff	S.Dev	Max
GidA	D		glucose-inhibited division protein	0445	23	.92	4.27	11.90
-	R		GTP-binding protein	0012	43	.94	3.84	12.43
RuvB	L		Holliday junction DNA helicase	2255	19	.89	4.32	12.51
Pnp	J		polynucleotide phosphorylase	1185	25	.86	4.94	13.11
PyrG	F		CTP synthetase	0504	26	.91	4.19	13.95
LepA	N		GTP-binding elongation factor	0481	15	.92	4.88	14.00
DnaX	L		DNA polymerase III subunits gamma and tau	2812	86	.90	4.37	14.59
Mfd	LK		transcription-repair coupling factor	1197	31	.88	4.82	14.94
UvrB	L		DNA excision nuclease subunit B	0556	12	.93	4.44	16.29
InfB	J		translation initiation factor IF-2	0532	32	.90	4.59	17.46
Exo	L		DNA polymerase I	0258	59	.89	4.85	17.60
PolC	L		DNA polymerase III, alpha chain	0587	61	.77	6.43	17.81
RecA	L		RecA protein	0468	4	.85	6.22	19.18
GyrA	L		DNA gyrase subunit A	0188	10	.88	5.60	19.74
HflB	0		cell division protein FtsH	0465	2	.86	5.29	19.89
ClpX	O		ATP-dependent Clp protease, ClpX	1219	13	.89	5.12	20.10
ThrS	J		threonyl-tRNA synthetase	0441	33	.77	6.94	20.19
Rho	K		transcription termination factor rho	1158	3	.87	5.83	20.20
GroL	O		GroEL, chaperone Hsp60	0459	8	.92	5.90	20.35
ClpB	0		ClpB protein	0542	7	.75	6.23	21.01
-	R		putative GTP-binding protein	1160	165	.94	7.93	21.07
DnaK	0		dnaK, chaperone Hsp70	0443	5	.81	6.48	21.27
RpSA	J		30S ribosomal subunit protein S1	0539	38	.88	8.14	22.48
RpoA	K		DNA-directed RNA polymerase alpha chain	0202	102	.91	10.93	32.41
TrxB	0		thioredoxin reductase	0492	66	.87	8.67	32.54
UvrC	L		excinuclease ABC subunit C	0322	133	.93	6.67	32.68
NusA	K		transcription pausing	0195	106	.83	11.11	39.39
QRI7	O		o-sialoglycoprotein endopeptidase	0533	123	.89	7.53	43.68
YidC	N		60 kD inner membrane protein	0706	179	.85	13.69	53.24
SecY	N		subunit of translocase	0201	82	.86	8.00	58.78

Methionyl-tRNA synthetase seems to have been horizontally transferred between Cyanobacteria and Firmicutes, isoleucy1-tRNA synthetase between Actinobacteria and Rickettsiales, and at least one domain of alanyl-tRNA synthetase between Aquifex and Bacteroidetes/Chlorobi. Researchers have been somewhat divided about tRNA synthetases. Brown et al. [5] used them as phylogenetic characters, but Brochier et al. [4] used them as a gene sample enriched in HGT. By individually screening protein families for representativeness for a specific set of organisms, we can use the representative tRNA synthetases and avoid the anomalous ones; for example we use threonyl-tRNA synthetase (correlation coefficient .77) for the left half of Figure 1.

**Figure 2:**
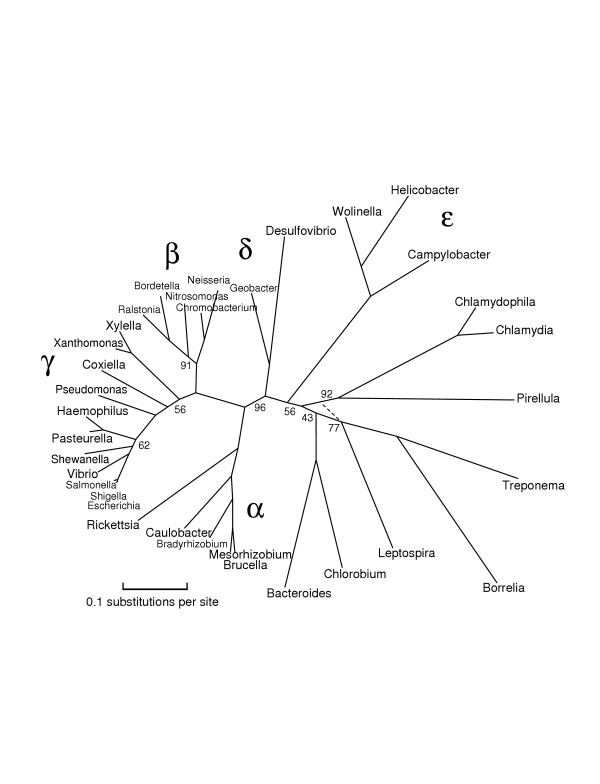
**An unrooted phylogenetic tree of Proteobacteria and related bacteria**. This tree shows the left half of Figure 1, including a number of additional genomes. The numbers associated with edges give bootstrap support as before; the best supported alternative choices are shown dashed. This tree slightly favors breaking the Spirochaetes/Chlamydiales clade of Figure 1.

**Figure 3:**
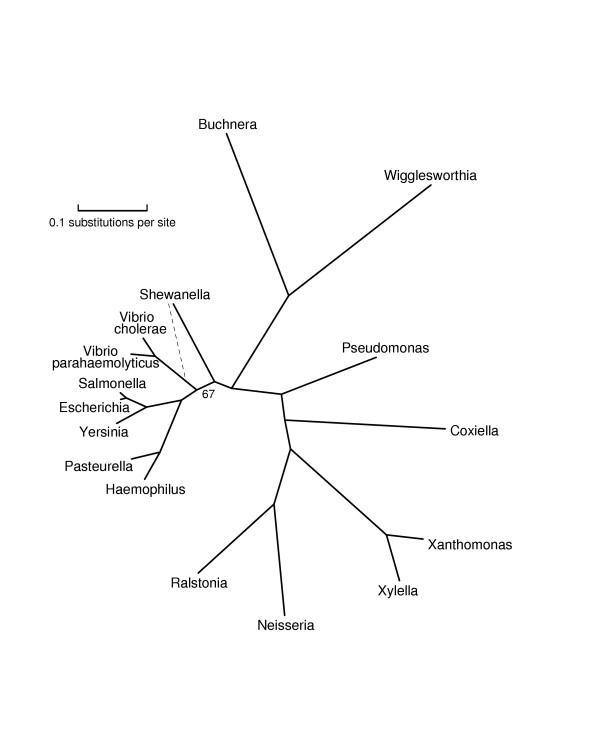
**An unrooted phylogenetic tree of *β*- and *γ*-Proteobacteria**. The edge labeled 67 is the only edge without bootstrap support of 100; the one alternative topology covers the other 33 bootstrap trials. This tree switches the branching order of Vibrio and Haemophilus from that shown in Figures 1 and 2. This tree should be more reliable, due to better taxon sampling and protein representativeness. For this less diverse set of taxa, many proteins had correlation coefficients greater than .90 with the consensus distance matrix. Notice that the two species of Vibrio are much more diverged than Escherichia and Salmonella.

**Figure 6:**
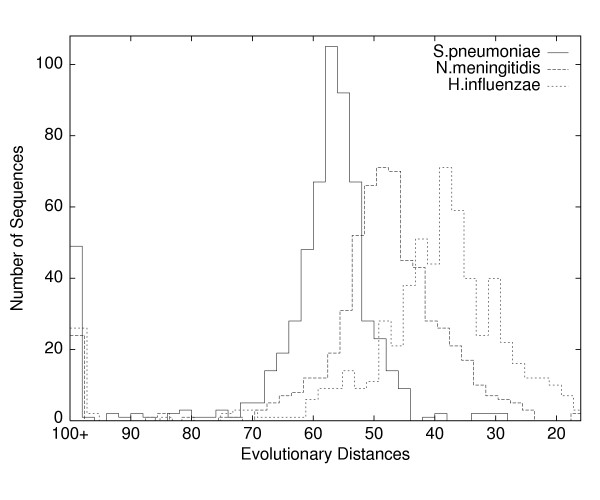
**Histograms of evolutionary distances**. Plotted are the evolutionary distances, between E. coli and three other bacteria, Streptococcus pneumoniae, Neisseria meningitidis, and Haemophilus influenzae. Each distance *D*(*i, j, k*), described in the Methods section, is given by a pairwise alignment of amino acid sequences of a given length (typically 300 residues), the most conserved subsequences for a family of orthologous proteins. We can interpret distances as times, with greater time towards the left. All three histograms are roughly bell-shaped but with rather high variances, which suggests that reliable phylogenetic inference requires either a great many sequences or representative sequences that sit near the center in all pairwise histograms. The peaks at 100+ indicate missing orthologs. There are several apparent horizontal transfers (right-side outliers) in S.pneumoniae and N.meningitidis. Even discounting the peaks at 100+, the left-side outliers (rapid evolution, large insertions or deletions, missing domains, hidden paralogs, and horizontal transfers from more distant organisms) outnumber right-side outliers; this pattern holds true even for very distant pairs such as S.pneumoniae and E.coli.

Despite the examples just given, representative proteins do tend to come disproportionately from core functional categories such as transcription and translation, consistent with the hypothesis [29,30] that HGT is less common for informational proteins than for metabolic proteins. Table 2 gives the list of representative proteins used to compute Figure 2. Several poorly characterized GTPases (Ffh, Obg, LepA, COG0012, COG1160), rarely used in phylogeny, repeatedly turned out to be representative [see Additional file 1]. Table 2 also lists three different measures of representativeness – agreement with the consensus distance matrix – for the representative proteins. One of these measures, the maximum elementwise difference between the single-gene and the consensus distance matrices, is generally quite large; every protein gave some pairwise distance that differed by at least 10% from the corresponding consensus distance. This observation means that very few proteins land near the center of all pairwise histograms (Figure 6) for a diverse set of bacteria; most proteins are well away from the mode in at least one such histogram. All measures of representativeness improve quite dramatically, however, with decreasing taxon diversity. The computation of Figure 3 for *β*- and *γ*-proteobacteria used only proteins with correlation coefficients at least .88, whereas the overall bacterial phylogeny necessarily used proteins with correlation coefficients as low as .62.

### Bacterial phylogeny

**Figure 4:**
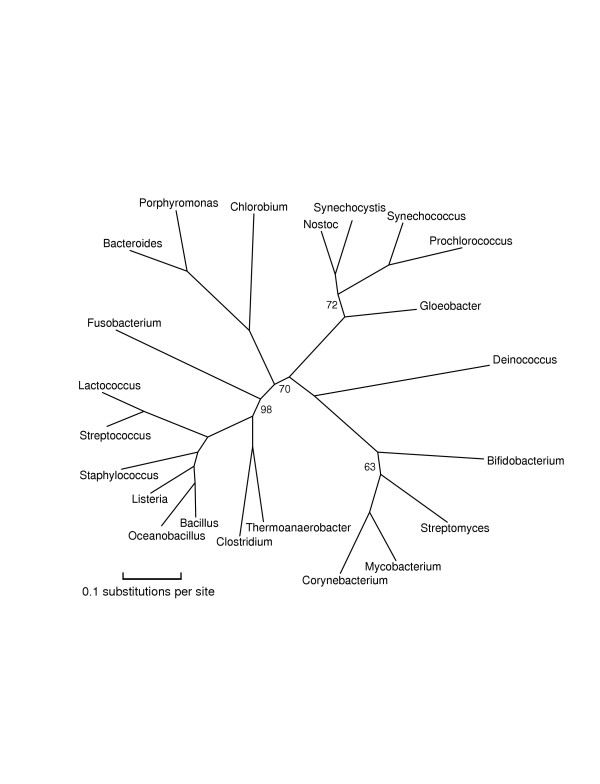
**An unrooted phylogenetic tree of Firmicutes, Actinobacteria, and Cyanobacteria**. Chlorobium and Bacteroides were included here and in Figure 2 in order to orient the trees relative to each other.

**Figure 5:**
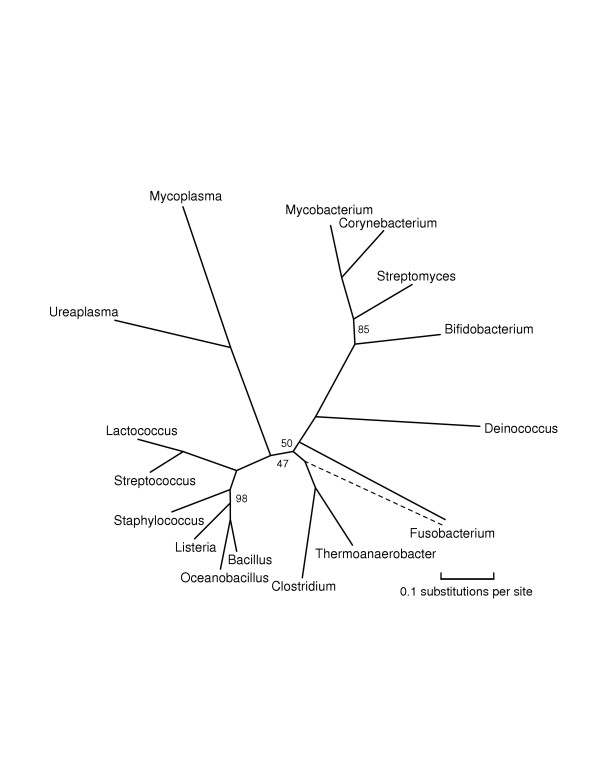
**An unrooted phylogenetic tree of Firmicutes and Actinobacteria**. This tree includes the reduced genomes of Mycoplasma and Ureaplasma. We found that if these genomes were included in larger phylogenies, such as those in Figures 1 and 4, we obtained unreliable results with poor bootstrap support.

Figures 1, 2, 3, 4, 5 give our phylogenies for Bacteria. These trees provide further validation of the methodology in the sense that they correctly identify all accepted clades, and are fairly robust under bootstrapping and under varying the choices of representative proteins and species. These trees were computed by maximum-likelihood methods on alignments of 5000–8000 columns, concatenations of (typically 300-residue) subsequences of the 20–40 most representative proteins for the set of genomes under study. The same number – or even a greater number – of columns of randomly chosen ubiquitous proteins gives worse results, not always finding the monophyly of Proteobacteria and of *γ*-Proteobacteria, two well-accepted clades that are nontrivial to resolve [23]. As explained in the Methods section, Figure 1 was computed all at once with a subsample of organisms, and again in two overlapping pieces (which gave compatible trees), each piece again using 5000–8000 columns. A separate run with the same subsample of organisms along with two archaea (Aeropyrum and Methanopyrus) was used to root the tree, and yet another separate run was used to place Chloroflexus, a partially sequenced genome. Figures 1, 2, 3, 4, 5 are in almost complete agreement with each other, as were the several different runs used to compute Figure 1, so our results seem to be robust under different taxon samples. One exception is Bacteroidetes/Chlorobi; if this phylum is omitted as in [6] deep branches rearrange, for example, Actinobacteria/Deinococcus/Cyanobacteria forms a high-level clade with Firmicutes. Figure 1 suggests that Chlorobium is the organism that has diverged least from the difficult-to-resolve central area of the tree.

The trees given in Figures 1, 2, 3, 4, 5 show substantial areas of agreement with those of other researchers [4-7,17]. Especially striking is the agreement with the recently published phylogeny by Battistuzzi et al. [17], computed from about 8000 columns from 32 manually selected genes. Figure 1 disagrees with the tree of Battistuzzi et al. only at three internal branches with poor bootstrap support in both studies: the root (they use the dashed edge), the edge marked 81 (they join Actinobacteria/Deinococcus/Cyanobacteria with Firmicutes), and the edge marked 85 between the branches to Haemophilus and Vibrio (they switch the order as in our Figures 2 and 3).

Our study supports a basal position for Aquifex as in [4,6,17], rather than placing it near Bacteroidetes/Chlorobi [7,8] or Proteobacteria [31]. We place Bacteroidetes/Chlorobi as a bridge between Gram-positive bacteria and Proteobacteria as in [17] and in the rRNA tree of Brochier et al. [4]. We form a clade containing Actinobacteria, Deinococcus, and Cyanobacteria ("Terrabacteria" [17]) as in [4-6,17], but (just barely) reject a higher-level clade [6,17] combining Actinobacteria/Deinococcus/Cyanobacteria with Firmicutes. We place Planctomycetes (Pirellula) with Chlamydiales as in [32]. We find that Spirochaetes, Chlamydiales (plus Pirellula), and *ε*-Proteobacteria form a close trio, with exact branching order hard to resolve, due to closely spaced branching events and/or numerous horizontal transfers. The bootstrap values at the branches in this trio stand out as weak in our generally well-resolved tree. We also consider the root to be relatively weak, not only because of its bootstrap value but also because it appears next to two thermophilic eubacteria showing the most HGT with Archaea. Our program rejects obvious cases of HGT as non-representative, but it cannot screen out subtle ancient transfers or genome-wide biases in amino acid composition. We reject grouping *δ*- and *ε*-Proteobacteria into a clade as in GenBank's taxonomy, because such a grouping had zero support out of 100 trials. We place Buchnera/Wigglesworthia farther than Haemophilus, Vibrio, and Shewanella from E. coli, in agreement with [17], but in disagreement with a recent phylogeny of *γ*-Proteobacteria [33] and two more focused studies [34,35]. Our placement of Buchnera/Wigglesworthia, however, may be an artifact related to long-branch attraction as in problem 4 of Table 1, so the true position of these reduced genomes may indeed be with enterobacteria.

Our tree supports the history of photosynthetic organisms determined by signature sequences [8]; moreover it places Chloroflexus, Chlorobium, and Cyanobacteria in close proximity. Even so, our tree implies either HGT or a common ancestor with both RC-1 and RC-2 types of reaction centers [8,36,37]. One explanation of the paraphyly of RC-1 and RC-2 would be horizontal transfer from Chloroflexus/Cyanobacteria to Proteobacteria, and indeed our program flags protochlorophyllide reductase, both ChlB and ChlN subunits, as anomalously close between Cyanobacteria and photosynthetic proteobacteria. Our program does not, however, find an unusually large amount of HGT among photosynthetic bacteria.

## Discussion

Any attempt to screen proteins for anomalous evolution inevitably leads to questions concerning horizontal gene transfer. The rate of HGT is notoriously difficult to estimate [38,39]. One study [40] indirectly estimates that about 30% of genes have been subject to HGT within Bacteria, whereas a more direct analysis finds evidence for HGT in about 50% of ubiquitous genes over all prokaryotes [17]. A recent study [26] finds that about 30% of single-ortholog COGs deviated significantly from "clock-like evolution" over smaller taxa, such as *γ*-Proteobacteria, with about half of these deviations due to HGT and half due to other anomalies. In what is perhaps the most detailed study to date, Lerat et al. [33] found only two strong examples of horizontal transfers among 205 genes within *γ*-Proteobacteria.

We did not derive estimates of the rate of HGT from our experiments; however, our finding that representativeness improves dramatically with decreasing taxon diversity suggests that any study relying on a set of bacterial genomes from a single phylum, as in the recent work on *γ*-Proteobacteria [26,33], is likely to underestimate the rate of HGT. Our finding (Figure 6) that unusually large pairwise evolutionary distances are much more common than unusually small distances confirms [26] that many proteins deviate from clock-like evolution due to discontinuous evolutionary events besides HGT.

In a recent study, Raymond et al. [41] concluded that HGT has obscured the history of photosynthetic organisms on the basis of widespread disagreement among 188 gene trees relating five such organisms: Synechocystis, Chloroflexus, Chlorobium, Rhodobacter, and Heliobacterium (close to Clostridium). We think, however, that the phylogeny of these five diverse and sparsely sampled [42] bacteria is simply too hard a problem for single gene sequences to resolve. We found that 100 bootstraps from single genes also gave widespread disagreement among resulting trees. An important point is that the number of alignment sites (columns) required to build a robust tree depends not on the number of organisms but rather on the length of the shortest internal tree edge. Daubin et al. [43] recently made this same point, and further argued that HGT has been overused as an explanation for the difficulty of bacterial phylogeny. Horizontal transfer, however, at the rate of one core gene replaced – "xenologous displacement" [26] – in each genome per 100 million years would be sufficient to pose an obstacle to deep bacterial phylogeny, yet remain undetected by the technique of Daubin et al., which looked for transfers into exactly one of a closely related pair (probably less than 100 million years since divergence) such as E. coli and Salmonella typhimurium.

## Conclusion

Ultimately prokaryotic phylogeny faces many obstacles beyond horizontal transfer. Even in eukaryotes, where HGT is extremely rare, Rokas et al. [44] found that concatenations of a large number of genes gave more consistent trees and better bootstrap support than single genes and concatenations of fewer than 20 genes. Representative proteins are clock-like proteins, ubiquitous over the set of genomes under study, selected with reference to a distance matrix computed by robust statistical methods from hundreds of well-conserved, but not necessarily ubiquitous, genes. Concatenations of representative proteins give results consistent with much larger concatenations of randomly selected proteins for phylogenetic problems with known answers, so there is reason to believe that representative proteins can also provide the statistical leverage necessary to shed light on deep unresolved branches that remain controversial even with the use of all ubiquitous genes. The method of representative proteins, however, like all phylogenetic methods, has strengths and weaknesses. By incorporating information from many faster-evolving, non-ubiquitous genes, the method may be more susceptible to long-branch attraction and convergent evolution than reliance on ubiquitous genes.

In our view, genes have rich and varied evolutionary histories, contingent upon selective pressures and random events at several different levels. Hence genes are likely to show continuous variation in quality as phylogenetic characters, rather than falling into two universal categories, bad and good, "subject to horizontal transfer" and "conserved core" [4,19]. There may well be genes whose entire evolutionary histories are smooth and regular, but there are surely not enough of them to resolve all phylogenetic questions, and we will be forced to use other, locally reliable genes to resolve certain parts of the tree of life. Hence we believe that the tools we developed for mining and ranking potential characters fill an important niche in the evolving methodology of phylogenetic inference.

## Methods

We used the publicly available genomes from GenBank [45] and the Joint Genome Institute [46]. We relied on the annotations to identify protein-coding genes, but not to identify orthologous sequences. All the software developed for this project is available from the authors upon request.

### Orthologous sequences

We used our own data-mining program (manuscript in submission), similar to an all-against-all BLAST search [47], to find orthologous sequences and rank them by degree of conservation. Given a set of genomes and a sequence length, say 300 amino acid residues, this program returns a ranked list of the 200 most conserved proteins at that length, along with pointers into the genomes for the locations of the sequences. If there are not 200 well-conserved orthologous families within the set of genomes – for example if the set includes a mix of eubacteria and archaea – then the program returns only as many families as are deemed to be obvious homology (approximately 30% pairwise identity). The choice of 200 is somewhat arbitrary, but seemed to be close to the maximum for genome sets containing more than one bacterial phylum, without incurring much misalignment or mutational saturation. The program is not designed to find remote homology, and existing tools such as PSI-BLAST [48] are in fact better for this task. We ran the program for several different sequence lengths from 60 to 300, and included each representative protein only once, at the maximum length for which it was representative.

We could have used existing tools such as BLAST or existing databases such as COGs for ortholog assembly, but our own program offered several advantages. First, it ranks ortholog families by conservation level, measured by the quartile log odds similarity over all pairs, that is, the similarity score greater than 1/4 and smaller than 3/4 of the pairwise similarities. (Thus if a gene is missing from more than 1/4 of the genomes under study, it has a conservation level near zero.) The program limits attention to sequences of fixed length, such as 300 residues, in order to compare conservation levels fairly. Then by examining ortholog families in order of decreasing conservation level, the program screens out families that are not sufficiently conserved. Second, it is much faster than BLAST, so that we could perform ortholog assembly separately for each set of genomes, thereby finding proteins conserved within the taxa under study, but not conserved more generally. Third, because it finds ortholog families using all pairwise alignments of candidate orthologs, rather than by a reciprocal or circular BLAST search, it also minimizes the problem of hidden paralogy [49]. We rarely observed paralogs (as identified by GenBank annotations) when the sequence length was greater than 100 residues. Moreover, selection of representative proteins should filter out remaining hidden paralogy that could mislead the tree-building program.

### Representative sequences

The next step condenses the large amount of information in the conserved sequences to a matrix {**D**(*i*, *j*)} of pairwise evolutionary distances. The step also retains a matrix {*D*(*i, j, k*)} for each protein *k*, that is, the pairwise evolutionary distances given by the *k*-th set of orthologous sequences. The amount of agreement between {*D*(*i, j, k*)} and {**D**(*i*, *j*)} determines whether protein *k *is representative. We start by computing, for each pair of organisms and each protein, a pairwise alignment and a log odds score (Smith-Waterman algorithm using the BLOSUM50 substitution matrix with default gap costs). This gives a three-dimensional array of log odds similarity scores *S*(*i, j, k*), where *i *and *j *index the organisms and *k *indexes the proteins. There are many alternative choices of similarity scores, simpler scores such as percent identity and more complicated scores involving multiple BLOSUM matrices; the alternatives we tried gave nearly identical choices of representative proteins. We assume that each protein has its own "clock" as in Yang's proportional model [50], so that in any given time period, the log odds of a given amount of evolution for protein *k *is a fixed multiple of the log odds of the same amount of evolution for protein *k'*. We compute the multiplier *M*_*k *_for protein *k *with an iterative procedure. We first detect whether an organism *i *is missing protein *k*; for such an *i *and *k*, score *S*(*i, j, k*) is very low for all *j*. We discard these *S*(*i, j, k*) scores altogether. Then starting with *M*_*k *_= 1 for all *k*, we alternate the following steps. Convergence (to three decimal places) resulted after only three iterations.

1. For each *i *and *j*, set **S**(*i, j*) ← trimmed mean *M*_*k*_*S*(*i, j, k*). For each *i *and *j*, we drop the top and bottom 20% of the *M*_*k*_*S*(*i, j, k*) values and compute the mean of the middle 60%. We chose this trimmed mean after estimating the size of distribution tails (anomalous distances) in histograms such as those shown in Figure 6.

2. For each *k*, set *M*_*k *_← median **S**(*i, j*) / *S*(*i, j, k*). Then normalize *M*_*k *_← *M*_*k*_·*c*, where *c *= *m */ ∑_*k*_*M*_*k *_and *m *is the number of proteins, so that the *M*_*k*_'s average 1.0.

Step 1 forms a consensus over proteins and step 2 forms a consensus over organism pairs. We used robust statistics (trimmed means and medians) instead of ordinary means due to the many outliers. Multipliers typically varied from about 0.5 for the most conserved protein to about 1.6 for the 200-th most conserved protein. Novichkov et al. [26] independently developed a similar procedure; however, they used the median rather than trimmed mean in Step 1 and simply ran the two steps once each, without iteration. Because the consensus similarities ***S***(*i, j*) improve with estimates of the multipliers *M*_*k*_, iteration gives better results, as we discuss below.

We convert the scaled similarity scores *M*_*k*_·*S*(*i, j, k*) to evolutionary distances *D*(*i, j, k*) = *C *- log (*M*_*k*_·*S*(*i, j, k*)), where *C *is a constant chosen so that all the *D*(*i, j, k*) are positive. In the case that organism *i *or *j *was identified as missing protein *k*, we set *D*(*i, j, k*) = ∞. Figure 6 shows histograms of *D*(*i, j, k*) values for three different pairs of organisms; the distances for missing proteins appear at 100+.

We set the consensus distance **D**(*i, j*) between organisms *i *and *j *to be the trimmed mean (again using the middle 60%) of the finite (not from missing proteins) *D*(*i, j, k*) values. We tested how well distance matrices conformed to trees by running the Fitch-Margoliash algorithm [1,51] (program FITCH in the PHYLIP package [52]). For example, the Fitch-Margoliash tree [see Figure S1 in Additional file 1] gives 0.199 relative squared errror, that is, 0.199 = ∑_*i, j *_(**T**(*i, j*) - **D**(*i, j*))^2^/**D**(*i, j*)^2^, where **T**(*i, j*) is the pairwise distance given by the tree, and gives 1.51% average percent standard deviation (APSD) [51,52], a measure of the typical error of **T**(*i, j*) relative to **D**(*i, j*). For comparison, we tested the median procedure of Novichkov et al. by computing consensus similarities with **S**_*N*_(*i, j*) = median *S*(*i, j, k*) and consensus distances with **D**_*N*_(*i, j*) = *C *- log **S**_*N*_(*i, j*). On 8 out of the 9 distance matrices used in our study, {**D**(*i*, *j*)} conformed to tree distances (computed with FITCH) more closely than did {**D**_*N*_(*i, j*)}, with APSDs ranging from 0.63 to 3.67 for {**D**(*i, j*)} and from 0.92 to 4.26 for {**D**_*N*_(*i, j*)}. For the organisms in Figure S1 the FITCH tree produced from {**D**_*N*_(*i, j*)} broke several accepted clades and achieved a relatively poor APSD of 4.24%. The one case on which {***D***_*N*_(*i, j*)} outperformed {**D**(*i, j*)} was the taxon sample that included the partially sequenced genome Chloroflexus.

The distances {*D*(*i, j, k*)} given by a protein *k *can be regarded as a vector with *N *= *n*(*n *- 1)/2 entries, where *n *is the number of organisms. We computed three different measures of how well {*D*(*i, j, k*)} represents the consensus matrix {**D**(*i, j*)}. The three measures are: (1) correlation coefficient of {*D*(*i, j, k*)} with {**D**(*i, j*)}; (2) standard deviation, that is, ; and (3) max distance, that is, max_*i, j *_|*D*(*i, j*, *k*) - **D**(*i, j*)|. We chose the top-ranking proteins by max distance, enough proteins to give approximately 10,000 columns; we marked the worst 20% of proteins by each of measures (1) and (2), and dropped all marked proteins, thereby obtaining 6000–8000 columns. Thus a protein had to be good on all three measures to be considered representative; in particular, a protein had to be ubiquitous over the organisms under study. For this reason, we initially left out organisms with very reduced genomes, such as Mycoplasma and Buchnera; these organisms are best added to the tree (Figures 3 and 5) later using representative proteins chosen for a smaller range of taxa.

### Multiple alignment and tree building

We used CLUSTAL W [53] to compute a multiple alignment for each set of orthologs. We removed columns of possibly incorrect alignment using a method recommended by Sidow (personal communication). We first removed all columns containing a gap character -, and then removed columns to the left and right of a gap column until reaching a column of chemical agreement. A column of chemical agreement is one in which all the amino acids in the column are from a single group, where the groups are acidic residues {D, E}, aromatic residues {F, W, Y}, basic residues {H, K, R}, cysteine {C}, nonpolar residues {A, G, I, L, P, V}, and polar residues {M, N, Q, S, T}. We also removed blocks of more than eight consecutive columns in which no column was one of chemical agreement; even if such a highly variable block is a correct alignment (one-for-one amino acid substitution over evolutionary time), it may have too many superimposed mutations to be helpful for phylogeny. Finally we concatenated all the cleaned alignment files, obtaining a multiple alignment of about 5000–7000 columns.

We used SEMPHY Version 0.9 to compute phylogenetic trees [54]. SEMPHY is a relatively fast EM (expectation maximization) program for ML (maximum likelihood) phylogeny, which assumes a Markov model of evolution. We used the JTT model [55], which is SEMPHY's default. The newer SEMPHY Version 1.0 models mutation rate variation among columns with a discrete gamma distribution, but we found only insignificant differences in the resulting ML trees, so we preferred the simpler, homogeneous-rate model. Cleaned alignments of representative proteins tend to be more homogeneous than alignments of random proteins. To estimate the confidence of clades, we used a bootstrapping procedure or – more precisely – a jackknife procedure. To create a random subsample of the entire alignment file we included each block of 80 consecutive columns in the subsample with probability 0.5; this gives a slightly harsher test than the standard bootstrap. We tested one tree (the overall tree for Figure 1) both ways; the jackknife numbers were all 0–10% smaller than the corresponding bootstrap numbers. The rationale for randomly sampling blocks rather than individual columns was to effectively vary the set of representative proteins as in [44]. For each ML tree computation, we created 100 random subsamples and ran SEMPHY on each subsample. Typical running times were 15–20 minutes per subsample, or about 30 hours for 100 subsamples. We also tried PhyML [56], another fast program for ML phylogeny. With a single substition rate category, PhyML Version 2.4.4 is faster than SEMPHY (7 minutes versus 17 minutes for a computation with 29 organisms and 5424 columns). On the other hand, PhyML may be more prone to converge to a suboptimal local optimum, as we sometimes obtained a greater likelihood within PhyML by inputting SEMPHY's solution as PhyML's starting tree.

To produce Figure 1, we made five different runs of tree computations with different sets of genomes. We chose representative proteins separately for each run, because representativeness depends upon the set of genomes under study. An initial run with 30 diverse bacteria [the 30 bacteria shown in Figure S1 of Additional file 1], with all phyla except Planctomycetes represented, sketched out an unrooted phylogeny. The root was placed by a run with the same 30 eubacteria along with two archaea, Aeropyrum and Methanopyrus. We then split the tree in two pieces ("left" and "right") by cutting it at the deep interior edge with best bootstrap support, the doubled edge shown in the figure. Computing a large phylogenetic tree in pieces allows the use of more proteins of greater representativeness, but such a split must be done very cautiously because each organism potentially affects all the unknown sequences at interior nodes. We added more organisms, such as Xanthomonas and Vibrio, assigning them to the left or right half according to the accepted taxonomy. We then added a right-half organism (Chlorobium) to the left half, and a left-half organism (Escherichia) to the right half, and made two more runs of tree computations, one for the left and one for the right. These runs corroborated the split by their placements of Chlorobium and Escherichia. In Figure 1 all edge lengths and bootstrap-support numbers refer to these last two runs, except for the numbers on the doubled edge and the root. (Edge lengths on the left and right sides of the tree are thus not strictly comparable as they refer to different proteins.) Finally, we added Chloroflexus by rerunning the overall computation, that is, 31 organisms in all. The draft genome, however, is missing many typically representative proteins such as GroEL and elongation factor TU, so we could harvest only about 1200 columns of representative proteins, and we indicate its position by a dashed edge without bootstrap support. (The 84 in Figure 1 is the support for the Actinobacteria/Deinococcus/Cyanobacteria clade.) In order to produce Figure 2, we reran the organisms of the left half along with some other fully sequenced Proteobacteria, Spirochaetes, and Chlamydiales. For Figure 3, we reran our procedures on *γ*-and *β*-proteobacteria, now including the reduced genomes of Buchnera and Wigglesworthia. For Figure 4, we reran most of the organisms of the right half along with some other genomes, and finally for Figure 5 we ran Actinobacteria and Firmicutes (along with Fusobacterium and Deinococcus), now including the reduced genomes of Mycoplasma and Ureaplasma. We attempted to include these reduced genomes at the higher level of Figure 4, but obtained inconsistent results depending upon the proteins selected.

## Authors' contributions

DG wrote the program that assembles the orthologs and ranks them by degree of conservation. MB wrote the program that selects representative proteins, and carried out all the computational experiments.

**Table 3:** Bacterial genomes used in this paper. All phyla in GenBank as of December 2004 are represented. Bold letters give abbreviations used in Table 1.

Specific Genome	Taxonomy (Phylum; Class)
Corynebacterium glutamicum	Actinobacteria;Actinobacteria
**Mycob**acterium tuberculosis H37Rv	Actinobacteria;Actinobacteria
**Bifid**obacterium longum	Actinobacteria;Actinobacteria
Streptomyces avermitilis	Actinobacteria;Actinobacteria
Aquifex aeolicus	Aquificae;Aquificae
**Bac**teroides thetaiotaomicron VPI-5482	Bacteroidetes/Chlorobi;Bacteroidetes
**Chlor**obium tepidum TLS	Bacteroidetes/Chlorobi;Chlorobi
Porphyromonas gingivalis W83	Bacteroidetes/Chlorobi;Chlorobi
Chlamydia trachomatis	Chlamydiae/Verrucomicrobia;Chlamydiae
Chlamydophila pneumoniae AR39	Chlamydiae/Verrucomicrobia;Chlamydiae
Chloroflexus aurantiacus	Chloroflexi;Chloroflexi
Gloeobacter violaceus	Cyanobacteria;Chroococcales
Synechococcus sp WH8102	Cyanobacteria;Chroococcales
**Syn**echocystis PCC6803	Cyanobacteria;Chroococcales
**Nos**toc sp	Cyanobacteria;Nostocales
**Pro**chlorococcus marinus MIT9313	Cyanobacteria;Prochlorophytes
Deinococcus radiodurans	Deinococcus-Thermus;Deinococci
Bacillus subtilis	Firmicutes;Bacilli
Oceanobacillus iheyensis	Firmicutes;Bacilli
**List**eria monocytogenes	Firmicutes;Bacilli
**Staph**ylococcus aureus subsp. aureus N315	Firmicutes;Bacilli
**Lact**ococcus lactis	Firmicutes;Bacilli
**Strep**tococcus pneumoniae R6	Firmicutes;Bacilli
**Clost**ridium tetani E88	Firmicutes;Clostridia
Thermoanaerobacter tengcongensis	Firmicutes;Clostridia
**Mycop**lasma	Firmicutes;Mollicutes
**Urea**plasma	Firmicutes;Mollicutes
Fusobacterium nucleatum	Fusobacteria;Fusobacteria
Pirellula sp	Planctomycetes;Planctomycetacia
**Caul**obacter crescentus	Proteobacteria;Alphaproteobacteria
Rhodopseudomonas palustris	Proteobacteria;Alphaproteobacteria
**Bru**cella melitensis	Proteobacteria;Alphaproteobacteria
Bradyrhizobium japonicum	Proteobacteria;Alphaproteobacteria
**Mesor**hizobium loti	Proteobacteria;Alphaproteobacteria
**Rick**ettsia conorii	Proteobacteria;Alphaproteobacteria
**Ral**stonia solanacearum	Proteobacteria;Betaproteobacteria
**Neiss**eria meningitidis Z2491	Proteobacteria;Betaproteobacteria
Chromobacterium violaceum	Proteobacteria;Betaproteobacteria
Bordetella pertussis	Proteobacteria;Betaproteobacteria
Nitrosomonas europaea	Proteobacteria;Betaproteobacteria
Coxiella burnetii	Proteobacteria;Gammaproteobacteria
**Esch**erichia coli K12	Proteobacteria;Gammaproteobacteria
**Haem**ophilus influenzae	Proteobacteria;Gammaproteobacteria
Pseudomonas aeruginosa	Proteobacteria;Gammaproteobacteria
Shigella flexneri 2a	Proteobacteria;Gammaproteobacteria
Shewanella oneidensis	Proteobacteria;Gammaproteobacteria
**Vib**rio cholerae	Proteobacteria;Gammaproteobacteria
Xanthomonas campestris	Proteobacteria;Gammaproteobacteria
**Xyl**ella fastidiosa	Proteobacteria;Gammaproteobacteria
**Desulf**ovibrio desulfuricans	Proteobacteria;delta/epsilon subdivisions
**Geo**bacter sulfurreducens	Proteobacteria;delta/epsilon subdivisions
**Campy**lobacter jejuni	Proteobacteria;delta/epsilon subdivisions
**Heli**cobacter pylori J99	Proteobacteria;delta/epsilon subdivisions
**Wol**inella succinogenes	Proteobacteria;delta/epsilon subdivisions
**Borr**elia burgdorferi	Spirochaetes;Spirochaetes
**Lep**tospira interrogans	Spirochaetes;Spirochaetes
**Trep**onema pallidum	Spirochaetes;Spirochaetes
Thermotoga maritima	Thermotogae;Thermotogae

## Supplementary Material

Additional File 1 (1) a phylogenetic tree computed directly from a distance matrix for 30 diverse bacteria, (2) the representative proteins used in all the phylogenetic tree computations, and (3) some probable horizontal transfers detected by the study. Click here for file
