# Supplementary material for: Automatic selection of representative proteins for bacterial phylogeny
Source: BMC Evol Biol. 2005 May 31;5:34. doi: 10.1186/1471-2148-5-34 (PMC1175084; doi:10.1186/1471-2148-5-34)
Supplement: Additional File 1 — (1) a phylogenetic tree computed directly from a distance matrix for 30 diverse bacteria, (2) the representative proteins used in all the phylogenetic tree computations, and (3) some probable horizontal transfers detected by the study. [file 1471-2148-5-34-S1.pdf]

Automatic selection of representative proteins for bacterial phylogeny – supplementary material

Marshall Bern*[1] and David Goldberg[1]

[1]Palo Alto Research Center, 3333 Coyote Hill Road, Palo Alto, CA 94304, USA

Email: Marshall Bern*- bern@parc.com; goldberg@parc.com;

*Corresponding author

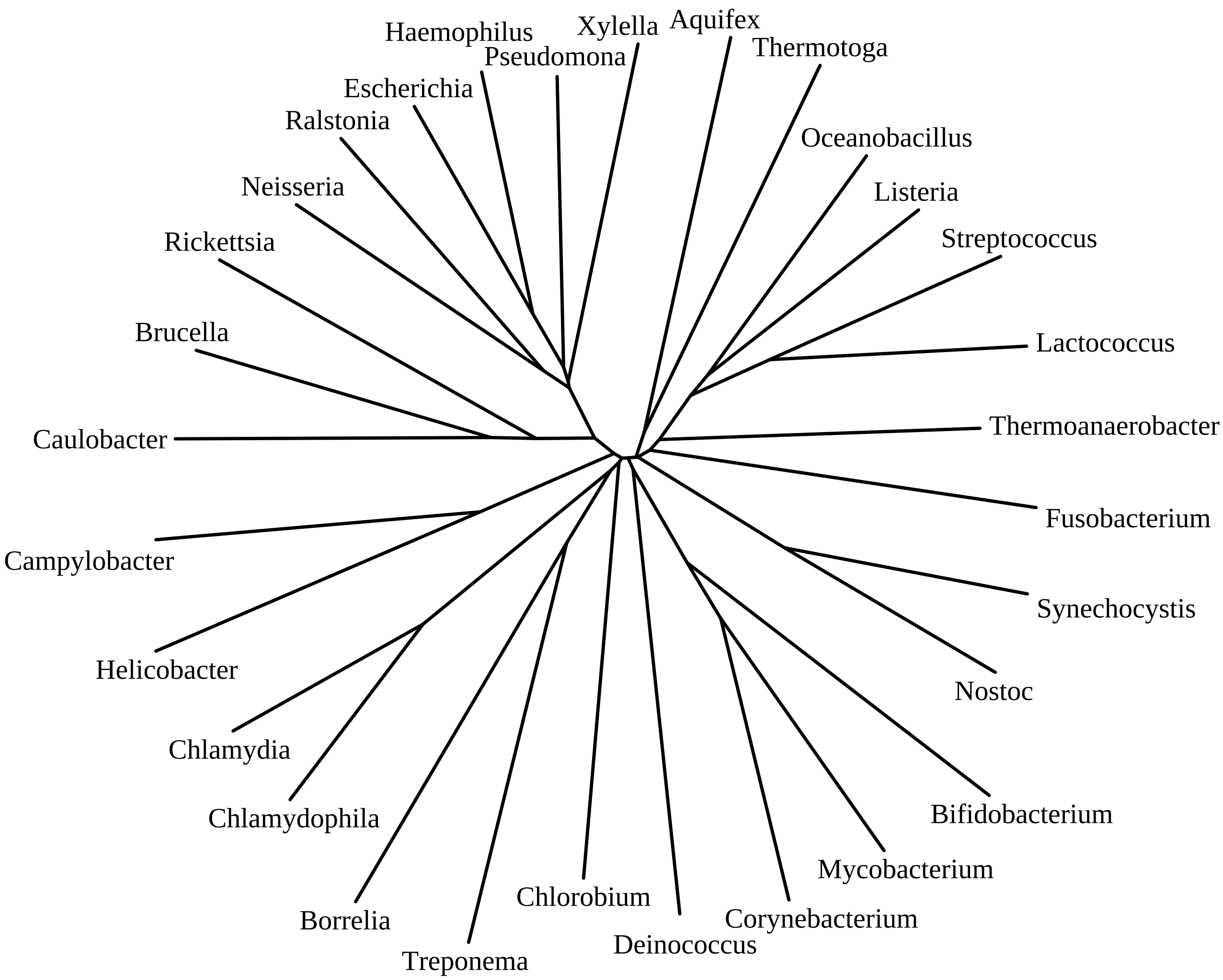

Figure S1: We computed a tree directly from a consensus distance matrix $\{\mathbf{D}(i,j)\}$ to test how well the matrix conformed with a tree metric. The consensus distance matrix used for this figure was computed from 200 families of orthologous sequences of length 300. The tree was computed using the Fitch-Margoliash algorithm (program FITCH from the package PHYLIP), and gave an average percent standard deviation of 1.51%, meaning that a typical pairwise distance in the tree deviates by 1.51% from the distance in the matrix. The tree correctly reconstructs accepted clades such as Proteobacteria and Actinobacteria, but shows poor resolution in the center. The 30 organisms in the tree above were the 30 organisms used in the initial overall computation of the tree in Figure 1.

Gene	Class	Name	COG	Rank	Coeff
Ffh	N	signal recognition particle protein	0541	51	.88
HflB	O	cell division protein FtsH	0465	0	.91
RplB	J	ribosomal protein L2	0090	18	.87
AspS	J	aspartyl-tRNA synthetase, discriminating	0173	31	.85
-	R	conserved hypothetical protein	0012	42	.90
Nth	L	endonuclease III	0177	113	.86
TufB	JE	elongation factor TU	0050	2	.85
QRI7	O	O-sialoglycoprotein endopeptidase, putative	0533	77	.89
LepA	N	GTP-binding elongation factor family protein LepA	0481	15	.89
InfB	J	initiation factor 2	0532	13	.86
FusA	J	elongation factor G	0480	12	.77
RpoC	K	DNA-directed RNA polymerase, beta‘ subunit	0086	2	.87
RpsS	J	ribosomal protein S19	0185	34	.83
RpsB	J	ribosomal protein S2	0052	63	.78

Table S1: Representative proteins used to root the bacterial tree of Figure 1. Sequence lengths in the first block are 180 and in the second block 60. The Rank column gives the conservation rank compared to other sequences of the same length; thus a 180-long sequence from elongation factor TU has rank 2 among 180-long sequences and a 60-long sequence from RNA polymerase, beta$'$ subunit, has rank 2 among 60-long sequences.

Gene	Class	Name	COG	Rank	Coeff
PrfA	J	peptide chain release factor 1	0216	31	.68
Ffh	N	signal recognition particle protein	0541	53	.85
GyrA	L	DNA gyrase, subunit A	0188	12	.76
InfB	J	initiation factor 2	0532	27	.81
FusA	J	elongation factor G	0480	15	.70
UvrB	L	excinuclease ABC, subunit B	0556	7	.79
RpoC	K	DNA-directed RNA polymerase, beta‘ subunit	0086	0	.67
ClpX	O	ATP-dependent Clp protease, ATP-binding subunit ClpX	1219	13	.76
-	R	conserved hypothetical protein	0012	38	.73
GroL	O	groEL protein	0459	8	.70
SecA	N	preprotein translocase, SecA subunit	0653	54	.64
ThrS	J	threonyl-tRNA synthetase	0441	39	.66
SecY	N	preprotein translocase, SecY subunit	0201	92	.66
QRI7	O	O-sialoglycoprotein endopeptidase, putative	0533	84	.81
-	R	conserved hypothetical protein	1160	116	.82
SerS	J	seryl-tRNA synthetase	0172	50	.65
DnaB	L	replicative DNA helicase	0305	86	.70
AlaS	J	alanyl-tRNA synthetase	0013	46	.72
RpoA	K	DNA-directed RNA polymerase, alpha subunit	0202	106	.72
Lig	L	DNA ligase	0272	70	.69
Pnp	J	polynucleotide phosphorylase	1185	23	.65
Obg	R	GTP-binding protein Obg	0536	35	.84
FtsY	N	signal recognition particle-docking protein FtsY	0552	71	.82
RecA	L	recA protein	0468	6	.63
HflB	O	cell division protein FtsH	0465	2	.79
RplB	J	ribosomal protein L2	0090	18	.70
DnaJ	O	dnaJ protein	0484	84	.80
TufB	JE	elongation factor TU	0050	4	.70
TatD	L	conserved hypothetical protein	0084	151	.74
RplE	J	ribosomal protein L5	0094	32	.82
RplF	J	ribosomal protein L6	0097	99	.71
Pth	J	peptidyl-tRNA hydrolase	0193	161	.72
RplC	J	ribosomal protein L3	0087	114	.73
Nth	L	endonuclease III	0177	149	.71
UvrC	L	excinuclease ABC, subunit C	0322	163	.71
MurA	M	UDP-N-acetylglucosamine 1-carboxyvinyltransferase	0766	86	.74
RplP	J	ribosomal protein L16	0197	36	.66
RplM	J	ribosomal protein L13	0102	70	.77
RplK	J	ribosomal protein L11	0080	38	.71
RpsE	J	ribosomal protein S5	0098	74	.62

Table S2: Representative proteins used to compute the initial, overall tree for Figure 1. Sequence lengths within the first block are all 300, second block 240, third block 160, and fourth block 130. Correlation coefficients are lower than in the other lists, because of the number and diversity of organisms. It is possible that almost all proteins have been horizontally transferred at some point within the history of Bacteria; in this case representative proteins are the ones with the least obvious—and hence least misleading—transfers.

Gene	Class	Name	COG	Rank	Coeff
GyrA	L	DNA gyrase, subunit A	0188	13	.88
LepA	N	GTP-binding elongation factor family protein LepA	0481	9	.88
DnaK	O	dnaK protein	0443	7	.87
GuaA	F	GMP synthase	0518	25	.87
RpoB	K	DNA-directed RNA polymerase, beta subunit	0085	11	.75
GroL	O	groEL protein	0459	8	.85
PrfA	J	peptide chain release factor 1	0216	52	.88
AspS	J	aspartyl-tRNA synthetase, discriminating	0173	38	.81
Obg	R	GTP-binding protein Obg	0536	51	.90
Eno	G	enolase	0148	15	.74
UvrB	L	excinuclease ABC, subunit B	0556	5	.72
DnaB	L	replicative DNA helicase	0305	105	.88
UvrA	L	excinuclease ABC, subunit A	0178	3	.75
RpoC	K	DNA-directed RNA polymerase, beta‘ subunit	0086	0	.70
ClpX	O	ATP-dependent Clp protease, ATP-binding subunit ClpX	1219	10	.79
FtsZ	D	cell division protein FtsZ	0206	40	.73
HflB	O	cell division protein FtsH	0465	4	.91
InfB	J	initiation factor 2	0532	26	.84
PrsA	FE	ribose-phosphate pyrophosphokinase	0462	53	.88
QRI7	O	O-sialoglycoprotein endopeptidase, putative	0533	99	.87
LysU	J	lysyl-tRNA synthetase	1190	35	.82
Sms	O	DNA repair protein	1066	78	.81
HflX	R	GTP-binding protein HflX	2262	104	.75
GpsA	C	glycerol-3-phosphate dehydrogenase, NAD(+)-dependent	0240	137	.81
TufB	JE	elongation factor TU	0050	1	.71
SerS	J	seryl-tRNA synthetase	0172	30	.79
RpoA	K	DNA-directed RNA polymerase, alpha subunit	0202	110	.76
DnaJ	O	dnaJ protein	0484	102	.78
-	R	conserved hypothetical protein	0012	59	.74
AlaS	J	alanyl-tRNA synthetase	0013	73	.80

Table S3: Representative proteins used to compute the left half of the tree in Figure 1. Lengths were all 300.

Gene	Class	Name	COG	Rank	Coeff
Ffh	N	signal recognition particle protein	0541	65	.95
-	R	GTP-binding protein, YchF family	0012	42	.94
Obg	R	GTP-binding protein CgtA	0536	67	.90
InfB	J	translation initiation factor IF-2	0532	32	.91
Exo	L	DNA polymerase I	0258	60	.91
LepA	N	GTP-binding protein LepA	0481	17	.92
GyrB	L	DNA gyrase subunit B	0187	18	.95
Pnp	J	polyribonucleotide nucleotidyltransferase	1185	20	.83
GidA	D	glucose inhibited division protein A	0445	21	.92
RuvB	L	Holliday junction DNA helicase RuvB	2255	23	.94
UvrB	L	excinuclease ABC, subunit B	0556	14	.95
PyrG	F	CTP synthase	0504	29	.89
LeuS	J	leucyl-tRNA synthetase	0495	37	.89
ClpX	O	ATP-dependent Clp protease, ATP-binding subunit ClpX	1219	15	.89
Rho	K	transcription termination factor Rho	1158	4	.91
HflB	O	cell division protein FtsH	0465	2	.86
GyrA	L	DNA gyrase subunit A	0188	10	.88
RecA	L	recA protein	0468	6	.86
-	R	GTP-binding protein	1160	147	.92
QRI7	O	peptidase M22 family protein	0533	108	.91
RpsA	J	ribosomal protein S1	0539	35	.88
SecY	N	preprotein translocase SecY subunit	0201	75	.83
RpoC	K	DNA-directed RNA polymerase, beta' subunit	0086	1	.89
GroL	O	chaperonin, 60 kDa	0459	8	.90
TufB	JE	translation elongation factor EF-Tu	0050	0	.85
AlaS	J	alanyl-tRNA synthetase	0013	27	.82
SerS	J	seryl-tRNA synthetase	0172	54	.79
Uup	R	ABC transporter, ATP-binding protein	0488	81	.87
Fmt	J	methionyl-tRNA formyltransferase	0223	120	.89
DnaG	L	DNA primase	0358	121	.90
RpoA	K	DNA-directed RNA polymerase, alpha subunit	0202	126	.91

Table S4: Representative proteins used to compute the right half of the tree in Figure 1. Lengths were all 300.

Gene	Class	COG	Name	Pair of Taxa
DnaA	L	0593	Chromosomal replication initiator	Actinobacteria - Firmicutes
RplD	J	0088	Ribosomal protein L4	Actinobacteria - Firmicutes
RplO	J	0200	Ribosomal protein L15	Actinobacteria - Firmicutes
MetG	J	0143	Methionyl-tRNA synthetase	Cyanobacteria - Firmicutes
SdhA	C	1053	Succinate dehydrogenase	Cyanobacteria - Proteobacteria
IlvD	EG	0129	Dihdroxy-acid dehydratase	Cyanobacteria - Proteobacteria
AvtA	E	0436	Aspartate aminotransferase	Cyanobacteria - Proteobacteria
TrmU	J	0482	tRNA methyltransferase	Lactobacillales - γ-Proteobacteria
Uup	R	0488	ABC transporter, EF-3 family	Actinobacteria - Proteobacteria
IleS	J	0060	Isoleucyl-tRNA synthetase	Actinobacteria - Rickettsiales
RpsN	J	0199	Ribosomal protein S14	Actinobacteria - Proteobacteria
FtsK	D	1674	Cell division protein FtsK	Spirochaetes - ϵ-Proteobacteria
Ndk	F	0105	Nucleoside diphosphate kinase	Chlamydiales - Proteobacteria

Table S5: Some proteins with anomalously small evolutionary distances between distant taxa, presumably due to horizontal transfer between ancestors. Notice that even "informational" proteins such as ribosomal proteins and tRNA synthetases are subject to horizontal transfer. Class refers to the COG (clusters of orthologous groups) functional codes given in Table S6.

	**Information storage and processing**
J	Translation, ribosomal structure and biogenesis
K	Transcription
L	DNA replication, recombination and repair
	**Cellular processes**
D	Cell division and chromosome partitioning
O	Posttranslational modification, protein turnover, chaperones
M	Cell envelope biogenesis, outer membrane
N	Cell motility and secretion
P	Inorganic ion transport and metabolism
T	Signal transduction mechanisms
	**Metabolism**
C	Energy production and conversion
G	Carbohydrate transport and metabolism
E	Amino acid transport and metabolism
F	Nucleotide transport and metabolism
H	Coenzyme metabolism
I	Lipid metabolism
Q	Secondary metabolites biosynthesis, transport and catabolism
	**Poorly characterized**
R	General function prediction only
S	Function unknown

Table S6: Functional categories of COGs.
